# The performance of screening tools and use of blood analyses in prehospital identification of sepsis patients and patients suitable for non-conveyance - an observational study

**DOI:** 10.1186/s12873-024-01098-4

**Published:** 2024-10-08

**Authors:** Agnes Olander, Lina Frick, Jennifer Johansson, Kristoffer Wibring

**Affiliations:** 1 Department of Health and Society, Kristianstad University, Kristianstad, Sweden; 2 PreHospen – Centre for Prehospital Research, University of Borås, Borås, Sweden; 3 Department of Ambulance and Prehospital Care, Region Halland, Sweden; 4 Institute of Health and Care Sciences, Sahlgrenska Academy, University of Gothenburg, Gothenburg, Sweden; 5 The Research Environment – Patient Reported Outcomes – Clinical Assessment Research and Education (PRO-CARE), Kristianstad University, Kristianstad, Sweden

**Keywords:** Emergency medical services, Prehospital, Ambulance services, Screening, Sepsis, Blood specimen, Blood test

## Abstract

**Background:**

Early recognition of sepsis by the EMS (Emergency Medical Services), along with communicating this concern to the emergency department, could improve patient prognosis and outcome. Knowledge is limited about the performance of sepsis identification screening tools in the EMS setting. Research is also limited on the effectiveness of prehospital use of blood tests for sepsis identification. Integrating blood analyses with screening tools could improve sepsis identification, leading to prompt interventions and improved patient outcomes.

**Aim:**

The aim of the present study is firstly to evaluate the performance of various screening tools for sepsis identification in the EMS setting and secondly to assess the potential improvement in accuracy by incorporating blood analyses.

**Methods:**

This is a retrospective observational cohort study. The data were collected from prehospital and hospital medical records in Region Halland. Data on demographics, vital signs, blood tests, treatment, and outcomes were collected from patients suspected by EMS personnel of having infection. The data were analysed using Student’s t-test. Sensitivity, specificity, positive predictive value, negative predictive value and odds ratio were used to indicate accuracy and predictive value.

**Results:**

In total, 5,405 EMS missions concerning 3,225 unique patients were included. The incidence of sepsis was 9.8%. None of the eleven tools included had both high sensitivity and specificity for sepsis identification. White blood cell (WBC) count was the blood analysis with the highest sensitivity but the lowest specificity for identifying sepsis. Adding WBC, C-reactive protein (CRP) or lactate to the National Early Warning Score (NEWS) increased the specificity to > 80% but substantially lowered the sensitivity.

**Conclusions:**

Identifying sepsis in EMS settings remains challenging, with existing screening tools offering limited accuracy. CRP, WBC, and lactate blood tests add minimal predictive value in distinguishing sepsis or determining non-conveyance eligibility.

**Supplementary Information:**

The online version contains supplementary material available at 10.1186/s12873-024-01098-4.

## Background

The Emergency Medical Services (EMS) are often the first healthcare contact for patients with infection and sepsis [1]. About 7% of all EMS missions involve patients with infection, and approximately 10% of these are diagnosed with sepsis [2]. Approximately 50–75% of all sepsis patients are assessed, cared for, and transported to the emergency department (ED) by the EMS [3–5].

Early identification of infection can prevent the development of sepsis, and early recognition of sepsis, along with communicating this concern to the ED, could improve patient prognosis and outcome [6, 7]. Previous studies have indicated that a documented suspicion of sepsis in the EMS records shortens the time to the administration of antibiotics [6, 8]. This is important since a delayed time to the start of antibiotics is associated with increased progression to septic shock and mortality [9]. However, research has indicated great variation in the identification of patients with sepsis (from 6 to 36%) in the EMS setting [6, 8, 10, 11].

There are several different screening tools that can be utilised in the EMS to identify sepsis. Some are specifically designed for sepsis [12–15], while others are more general triage tools [16, 17]. Assessing the accuracy of these screening tools is challenging. They were developed to predict various outcome measures, such as mortality, hospital admission, and the presence of sepsis. Furthermore, the definition and diagnostic criteria of sepsis have varied, particularly over time, which creates additional obstacles in evaluating the efficacy of these screening tools [18]. There are few large studies comparing more than individual tools within the same cohort [16, 17]. These screening tools were also developed prior to the emergence of COVID-19, and to the best of the authors’ knowledge, no studies have evaluated their efficacy in the context of COVID-19 becoming part of the normal medical spectrum.

Additionally, the role of prehospital blood tests such as lactate, White blood cell (WBC) count, and C-reactive protein (CRP) in enhancing sepsis identification is poorly understood. While elevated lactate levels are associated with worse outcomes [19–22], they have not consistently predicted sepsis in EMS settings, and studies on WBC count and CRP are scarce [23–27]. Further research is necessary to evaluate the performance of screening tools and the potential of blood analyses in improving the accuracy of sepsis identification and in determining the appropriateness of non-hospital referrals for infected patients. Understanding these factors could enhance early sepsis detection and decision-making in EMS care.

The aims of this study are:

- to evaluate the performance of different screening tools for the identification of sepsis in the EMS setting.
- to examine whether adding blood analyses improves sepsis identification accuracy.
- to investigate whether the National Early Warning Score (NEWS) along with WBC, CRP and lactate could be used for the identification of patients suitable for non-conveyance.

## Materials and methods

### Design

Retrospective observational cohort study.

### Study setting

Patients were recruited from the county of Halland, Sweden, with approximately 330,000 inhabitants and an area of 5,500 km^2^. Approximately 33,000 patient-related EMS missions are carried out annually (excluding transport between care facilities). During the day, approximately 20 ambulances operate and are staffed with at least one registered nurse each. There are two emergency hospitals, both with intensive care units. Ambulances are dispatched with priority 1–4, of which priority 1 is a suspected life-threatening condition. Priority 2 is urgent but not life-threatening. Priority 3 is cases for which ambulance transport is deemed necessary but not urgent. Priority 4 is transports without care needs but for which the patients need to lie down during transport [28]. Rapid Emergency Triage and Treatment System (RETTS) [16] is used for prehospital and emergency department triage. It has four different triage levels: red, orange, yellow and green. Red is the highest priority and indicates the need for immediate physician examination.

A non-conveyance protocol was in place during the data collection period. The design of the protocol has varied somewhat over time. However, decision on non-conveyance has mainly been based on the overall assessment of the EMS personnel rather than fixed directives. Patients who were not conveyed are not included in the current study.

### Data collection

The data were collected from 1 January 2019 until 31 December 2022.

### Inclusion criteria

- ≥ 18 years old.
- Infection susceptibility according to EMS personnel, i.e. Emergency Signs and Symptoms (ESS) code 44/47 according to RETTS.
- Patient transported to an ED in Region Halland.
- Ambulance dispatched with priority 1–3.
- Primary EMS mission, i.e. not transport from/between care facilities.

The data were collected via automated data extraction from the Region Halland data warehouse [29]. This technique for data extraction has been used previously with reliable results [30]. This data extraction identified 6,150 eligible EMS missions. A sample of data extracted from 20 EMS missions was manually compared to the original data source, i.e., prehospital and hospital medical records, to ensure data extraction quality. No inaccuracies were identified during the manual data check. Automated data extraction was used to extract data on the following variables:

EMS records:

- Patient age and sex.
- EMS mission priority.
- Vital signs.
- RETTS triage.
- Administration of oxygen and fluids.

Hospital records:

- ED antibiotic administration.
- ED blood analyses of WBC, CRP and lactate.
- Hospital admission.
- Intensive Care Unit (ICU) care.
- International Statistical Classification of Diseases and Related Health Problems (ICD) 10 diagnosis at the ED.
- ICD-10 diagnosis at hospital discharge.
- Mortality.

**Fig. 1:**
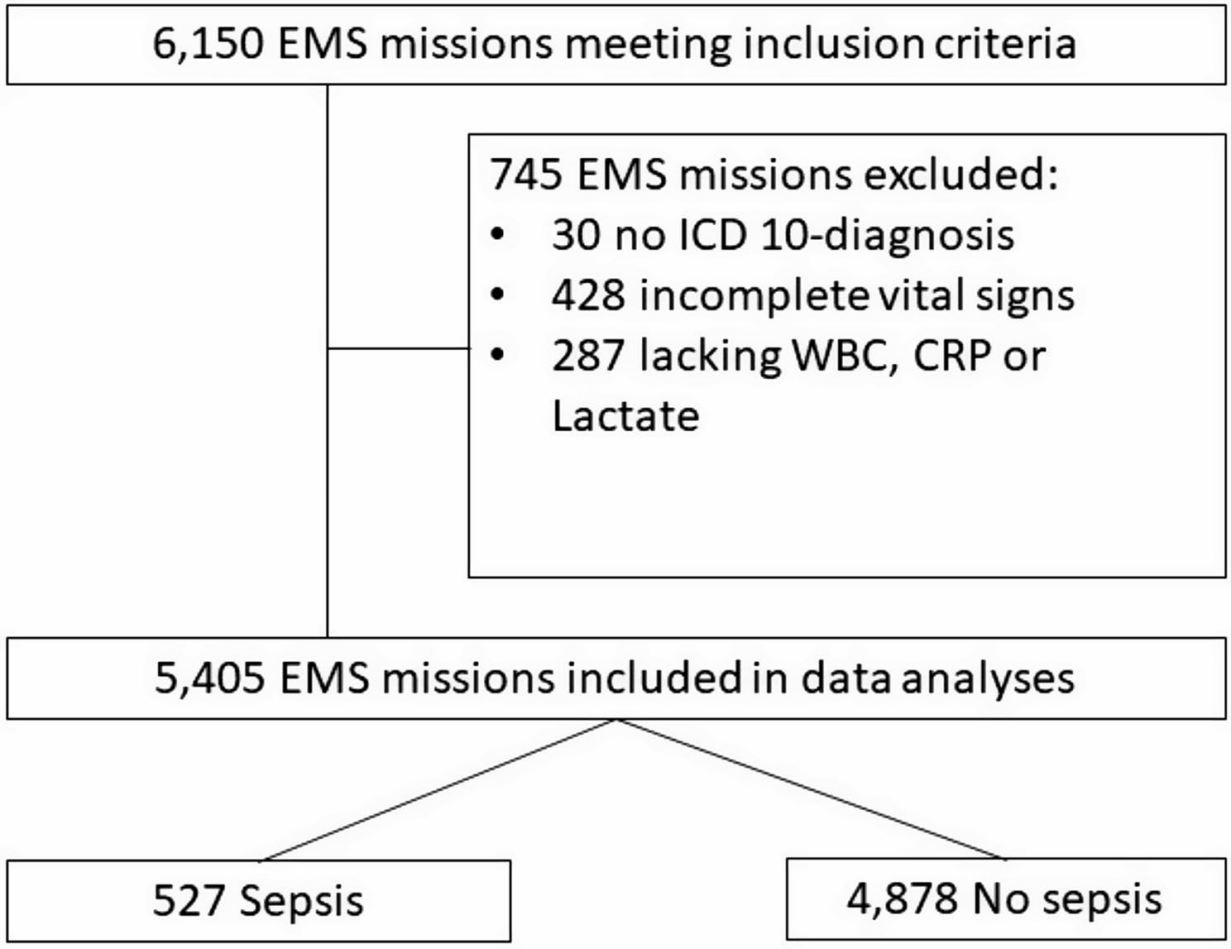
Study flow chart

EMS missions were excluded from the data analyses if there was no ICD-10 diagnosis, if the vital signs were incomplete, or if analysis of WBC, CRP or lactate was lacking at the ED (Fig. 1).

### Endpoints

- *Sepsis diagnosis during hospitalisation*. Defined as “sepsis” being included in the main ICD-10 diagnosed by the physician in charge at the ED or at hospital discharge. This included the following ICD-10 codes:
  - A327 - Listerial sepsis.
  - A400 - Sepsis caused by Group A streptococci.
  - A401 - Sepsis caused by Group B streptococci.
  - A402 - Sepsis caused by Group D streptococci and enterococci.
  - A403 - Sepsis caused by Streptococcus pneumoniae.
  - A408 - Other specified streptococcal sepsis.
  - A409 - Streptococcal sepsis, unspecified.
  - A410 - Sepsis caused by Staphylococcus aureus.
  - A411 - Sepsis caused by other specified staphylococci.
  - A412 - Sepsis caused by unspecified staphylococci.
  - A413 - Sepsis caused by Haemophilus influenzae.
  - A414 - Sepsis caused by anaerobic bacteria.
  - A415 - Sepsis caused by other Gram-negative organisms.
  - A418 - Other specified forms of sepsis.
  - A419 - Sepsis, unspecified.
  - B377 - Candidal sepsis.
  - In most cases, but not necessarily, sepsis diagnoses were based on a blood culture. The sepsis diagnoses were included in the endpoint regardless of whether covid-19 was present or not.
- *Suitable for non-conveyance*. Defined as patient being sent home directly from the ED without hospital admission.

### Sepsis screening tools

Eleven different screening tools were included. These were mainly based on vital signs but occasionally also included blood analyses, symptoms and age. Calculations of screening tool scores were based on the first set of registered vital signs from the EMS mission. Emergency Medical Dispatch Centre (EMDC) priority is solely based on symptoms (Table 1).

**Table 1:** Overview of included screening tools

Screening tool	RR	Oxygen saturation	SBP	HR	Level of consciousness	Temperature	Other	Positive score
**Sepsis specific**								
**Quick Sepsis-Related Organ Failure Assessment (qSOFA)** [12]	> 21		< 101		Altered			if ≥ 2 points
**Prehospital Sepsis Alert Protocol Criteria** [22]	> 20		< 90	> 90		> 38,0 or > 36,0	Lactate ≥ 4 mmol/L	if ≥ 2 points of RR, HR and temp and 1 point of SBT or Lactate
**PreSep score** [15]	> 22	< 92	< 90	> 90		> 38,0 or > 36,0		if ≥ 4 points
**Systemic Inflammatory Response Syndrome (SIRS)** [8]	> 20			> 90		> 38,0 or > 36,0	WBC > 12 × 10^9^/L or < 4 × 10^9^/L	if ≥ 2 points
**Modified SIRS** [31]	> 20			> 90		> 38,0 or > 36,0		if ≥ 2 points
**Blodtryck Andningsfrekvens Saturation (Swedish for Blood pressure**,** Breathing Rate**,** Saturation)** **(BAS) 90-30-90** [15]	> 30	< 90	< 90					if ≥ 1 points
**Prehospital Serve Sepsis (PRESS) score** [32]	> 20		< 110	> 90				if ≥ 3 points
**Non sepsis specific**								
**National Early Warning Score (NEWS)** [16]								if ≥ 7 points
**Rapid Emergency Triage and Treatment System (RETTS)** [16]	< 8 or > 30	< 87	< 90	> 130 or < 30	Unconscious		Specific symptoms	if ≥ 1 points
**Critical Illness Prediction (CIP) score** [17]	> 23 = 1 > 35 = 2		< 90 = 1	> 119 = 1	Altered = 1, Unconsciousness = 2		Age > 44 = 1	if ≥ 4 points = high risk
**Emergency Medical Dispatch Centre (EMDC) priority**							Specific symptoms	

WBC = White Blood Cell Count, SBP = Systolic blood pressure, RR = Respiratory Rate, HR = Heart Rate

NEWS have multiple thresholds for RR, oxygen saturation, SBP, HR, level of consciousness, and temperature. Therefore, specific thresholds have not been specified

### Blood analyses

The EMS in Region Halland do not have access to Point of Care Testing (POCT) for analyses of WBC, CRP or lactate. Data on WBC, CRP and lactate analyses were instead collected from the ED medical records. The potential added value of WBC, CRP and lactate levels was assessed if prehospital POCT was available. The first registered blood test from the ED was used, normally collected in conjunction with patients’ ED arrival.

There are no standardised cut-off values for WBC, CRP or lactate for sepsis detection. Cut-offs vary widely between different studies [33]. In the present study, the cut-off for WBC count was set to > 12 × 10^9^/L or < 4 × 10^9^/L in accordance with the System Inflammatory Response Syndrome (SIRS) criteria. The cut-off for CRP was set to > 90 mg/L in accordance with the study by Castelli et al. [34]. The cut-off for lactate was set to ≥ 4 mmol/L in accordance with the Prehospital Sepsis Alert Protocol Criteria [22].

Cut-offs for WBC, CRP and lactate for the identification of patients suitable for non-conveyance were based on reference intervals for WBC (3.5–8.8 4 × 10^9^/L) and lactate (0.5–2.2). A CRP concentration of < 10 mg/L was used as the cut-off.

### Data analysis

For descriptive analyses of categorical variables, frequencies and percentages are presented. Continuous variables are presented as medians and quartiles (Q1-Q3). Continuous variables were analysed with Student’s t-test. The accuracy and predictive value are described as sensitivity, specificity, positive predictive value (PPV), negative predictive value (NPV) and odds ratio. Logistic regression was used for calculating odds ratios. A p-value < 0.05 was considered statistically significant.

The potentially improved accuracy and predictive value of including WBC, CRP or lactate were examined by conducting analyses of the NEWS with and without these blood tests. The NEWS was chosen for these analyses as being a well validated, well known and widely used tool. Furthermore, it does not include any blood analyses, unlike some of the other tools included.

IBM SPSS Statistics for Windows version 29 (IBM Corp. Amonk, New York, USA) was used for all analyses.

## Results

In total, 5,405 EMS missions, concerning 3,225 unique patients were included. The median age was 79 years, and 59.5% of the EMS missions involved male patients. Almost half of the EMS missions were dispatched with priority 1 (lights and sirens). Prehospital intravenous fluids were administered in 49.8% of the cases. The corresponding figure for oxygen administration was 37.1%. Almost 90% of the patients were admitted to hospital, and 1.3% received intensive care. The thirty-day mortality rate was 14.1%. The incidence of sepsis was 9.8% (Table 2).

**Table 2:** Cohort characteristics in relation to sepsis and hospital admission

		Hospital admission		ICD-10 sepsis diagnosis		
	All % (*n*)	Hospital admission % (*n*)	ED discharge % (*n*)		Sepsis % (*n*)	Non-sepsis % (*n*)
**All**	100 (5405)	88.3 (4773)	11.7 (632)		9.8 (527)	90.2 (4878)
**Sex**						
**Men**	59.5 (3218)	88.9 (2862)	12.6 (274)		10.3 (333)	89.7 (2885)
**Women**	40.5 (2187)	87.4 (1911)	12.6 (276)		8.9 (194)	91.1 (1993)
**Age**,** median (Q25-Q75)**	79 (70–85)	79 (71–85)	75 (55.25-83)		79 (70–85)	79 (70–85)
**Priority out (EMDC)**						
**1**	47.8 (2584)	49.7 (2371)	33.7 (213)		63.2 (333)	46.1 (2251)
**2**	48.5 (2619)	46.8 (2236)	60.6 (383)		35.1 (185)	49.9 (2434)
**3**	3.7 (202)	3.5 (166)	5.7 (36)		1.7 (9)	4.0 (193)
**Priority in (EMS)**						
**1**	22.0 (1190)	24.5 (1171)	3.0 (19)		53.3 (281)	18.6 (909)
**2**	59.6 (3224)	58.6 (2796)	67.7 (428)		40.6 (214)	61.7 (3010)
**3**	17.7 (957)	16.4 (783)	27.5 (174)		6.1 (32)	19.0 (925)
**4**	0.6 (34)	0.5 (23)	1.7 (11)		−	0.7 (34)
**Prehospital oxygen administration**	37.1 (2006)	40.3 (1922)	13.3 (84)		55.6 (293)	35.1 (1713)
**Prehospital intravenous fluid**	49.8 (2691)	51.9 (2476)	34.0 (215)		64.9 (342)	48.2 (2349)
**Antibiotics administration in the ED**	55.2 (2981)	60.7 (2897)	13.3 (84)		87.1 (459)	51.7 (2522)
**Antibiotics administration in the ED (536 patients with Covid-19 excluded)**	58.8 (2865)	64.5 (2783)	14.8 (82)		88.3 (451)	55.4 (2414)
**Time (in minutes) between ED arrival and antibiotics administration**,** median (Q25-Q75)**	96 (47–153)	95 (46–150)	145 (90.5-223.75)		56 (32–106)	103 (53–160)
**ED blood analysis**						
**WBC x10**^**9**^**/L**,** median (Q25-Q75)**	10 (8–15)	11 (8–15)	9 (7–11)		12 (8–17)	11 (8–15)
**CRP mg/L**,** median (Q25-Q75)**	66 (25–130)	73 (30–137)	29 (9–62)		101 (42–176)	63 (24–124)
**Lactate mmol/L**,** median (Q25-Q75)**	7 (1.2–2.3)	1.7 (1.3–2.4)	1.4 (1.1–1.9)		2.5 (1.7–3.6)	1.6 (1.2–2.2)
**Admitted to hospital**	88.3 (4773)	100 (4773)	0 (0)		99.6 (525)	87.1 (4248)
**Intensive care unit care**	1.3 (69)	1.4 (69)	0 (0)		7.0 (37)	0.7 (32)
**Seven-days mortality (based on 3 225 unique patients)**	4.9 (157)	15.3 (149)	1.9 (8)		16.6 (52)	3.6 (105)
**Thirty-days mortality (based on 3 225 unique patients)**	14.1 (455)	15.6 (436)	4.5 (19)		31.6 (99)	12.2 (356)

EMDC = Emergency medical dispatch centre, EMS = Emergency medical service, ED = Emergency department, EMD = Emergency medical dispatch, WBC = White Blood Cell Count, CRP = C-Reactive Protein

The other potential infections included the following five most common diagnoses at hospital discharge after patient admission: J159 – Bacterial pneumonia, unspecified (10%); U071 – COVID-19, virus identified (9%); N109 – Acute tubulo-interstitial nephritis (8%) and N390 – Urinary tract infection, site not specified (8%); and T835 – Infection and inflammatory reaction due to internal prosthetic devices, implants and grafts in the urinary system (5%). Also included were several non-infectious diagnoses such as heart failure, pulmonary embolism, stroke and different sorts of tumour. A total of 536 patients (10%) were diagnosed with COVID-19 either at the ED or at hospital discharge.

Antibiotics were administered at the ED in 55.2% of the cases and the median time between ED arrival and ED administration was 96 min. For sepsis patients, the corresponding numbers were 87.1% and 56 min, respectively. When comparing patients who were diagnosed with sepsis and triaged red according to RETTS (highest triage colour) to patients with sepsis and a lower triage colour, the mean antibiotic administration times were 47 and 111 min, respectively, i.e. a difference of 64 min (*p* < 0.001, Student’s t-test).

### Identification of patients with sepsis

The accuracy of the different screening tools differed substantially. None of the tools had both high sensitivity and specificity. The sensitivity ranged from 24.1% (Prehospital Severe Sepsis (PRESS) score) to 85.4% (SIRS). The corresponding percentages for specificity were 35.2% (SIRS) and 94.7% (Prehospital Sepsis Alert Protocol Criteria). Blodtryck Andningsfrekvens Saturation (Swedish for Blood pressure, Breathing Rate, Saturation) (BAS) 90-30-90, the NEWS, the RETTS and EMDC dispatch priority were the only tools with a sensitivity and specificity above 50% (Table 3).

**Table 3:** Accuracy of screening strategies for prehospital identification of sepsis

	Sensitivity (CI 95%)	Specificity (CI 95%)	PPV (CI 95%)	NPV (CI 95%)	Odds ratio (CI 95%)
**Sepsis specific**					
Quick Sepsis-Related Organ Failure Assessment (qSOFA)	44.0 (40.0-48.4)	84.3 (83.2–85.3)	23.2 (21.2–25.4)	93.3 (92.8–93.8)	4.2 (3.5–5.1)
Prehospital Sepsis Alert Protocol Criteria	26.9 (23.2–31.0)	94.7 (94.0-95.3)	35.2 (31.2–39.5)	92.3 (91.9–92.7)	6.5 (5.2–8.2)
PreSep score	27.1 (23.4–31.2)	90.2 (89.3–91.0)	23.0 (20.3–26.1)	92.0 (91.6–92.4)	3.4 (2.8–4.2)
Systemic Inflammatory Response Syndrome (SIRS)	85.4 (82.1–88.3)	35.2 (33.9–36.6)	12.5 (12.0-12.9)	95.7 (94.8–96.5)	3.2 (2.5–4.1)
Modified SIRS	84.6 (81.3–87.6)	31.9 (30.6–33.2)	11.8 (11.4–12.3)	95.1 (94.0-95.9)	2.6 (2.0-3.3)
Blodtryck Andningsfrekvens Saturation (Swedish for Blood pressure, Breathing Rate, Saturation) (BAS) 90-30-90	52.0 (47.6–56.3)	75.8 (74.6–77.0)	18.8 (17.4–20.4)	93.6 (93.0-94.1)	3.4 (2.8–4.1)
Prehospital Serve Sepsis (PRESS) score	24.1 (20.5–28.0)	93.9 (93.1–94.5)	29.7 (26.0-33.8)	92.0 (91.6–92.3)	4.8 (3.8–6.1)
**Non sepsis specific**					
National Early Warning Score (NEWS) - ≥7 points	69.3 (65.1–73.2)	64.7 (63.3–66.0)	17.5 (16.5–18.5)	95.1 (94.5–95.7)	4.1 (3.3-5.0)
Rapid Emergency Triage and Treatment System (RETTS) - Red triage	56.6 (52.2–60.8)	77.0 (75.8–78.2)	21.0 (19.5–22.5)	94.3 (93.7–94.8)	4.4 (3.6–5.2)
Critical Illness Prediction (CIP) score	24.1 (20.5–28.0)	94.5 (93.8–95.1)	32.1 (28.1–36.4)	92.0 (91.7–92.4)	5.4 (4.3–6.9)
Emergency Medical Dispatch Centre (EMDC) highest priority	63.2 (58.9–67.3)	53.9 (52.4–55.3)	12.9 (12.1–13.7)	93.1 (92.4–93.8)	2.0 (1.6–2.4)
WBC > 12 × 10^9^/L or < 4 × 10^9^/L	63.2 (58.9–67.3)	54.4 (53.0-55.8)	13.0 (12.2–13.9)	93.2 (92.4–93.9)	2.1 (1.7–2.5)
CRP > 90 mg/L	55.6 (51.2–59.9)	62.9 (61.5–64.2)	13.9 (12.9–15.0)	92.9 (92.2–93.5)	2.1 (1.8–2.5)
Lactate ≥ 4 mmol/L	21.6 (18.2–25.4)	95.9 (95.4–96.5)	36.54 (31.8–41.6)	91.9 (91.6–92.2)	6.5 (5.1–8.4)
≥ 7 points NEWS and WBC > 12 × 10^9^/L or < 4 × 10^9^/L	45.0 (40.7–49.3)	81.7 (80.6–82.8)	21.0 (19.2–22.9)	93.2 (92.7–93.7)	3.7 (3.0-4.4)
≥ 7 points NEWS and CRP > 90 mg/L	37.4 (33.2–41.7)	85.9 (84.9–86.8)	22.2 (20.0-24.5)	92.7 (92.2–93.1)	3.6 (3.0-4.4)
≥ 7 points NEWS and lactate ≥ 4 mmol/L	17.3 (14.1–20.8)	97.5 (97.1–98.0)	43.1 (37.0-49.5)	91.6 (91.3–91.9)	8.3 (6.2–11.1)
≥ 7 points NEWS and WBC > 12 × 10^9^/L or < 4 × 10^9^/L, CRP > 90 mg/L or lactate ≥ 4 mmol/L	59.4 (55.1–63.6)	75.3 (74.0-76.5)	20.6 (19.2–22.1)	94.5 (93.9–95.0)	4.5 (3.7–5.4)

CI = Confidence Interval, PPV = predictive value, NPV = Negative predictive value, WBC = White Blood Cell Count, CRP = C-Reactive Protein

### Added value of prehospital blood analyses

The WBC was the blood analysis with the highest sensitivity, 63.2%, but the lowest specificity, 54.4%, for sepsis identification. Lactate had the opposite effect, with a sensitivity of 21.6% and a specificity of 95.9%. Adding WBC, CRP or lactate to the NEWS increased specificity to > 80% but substantially lowered sensitivity. The combination of NEWS and lactate reached a specificity of 97.5%, but sensitivity decreased to 17.3% (Table 3).

### Identification of patients suitable for non-conveyance

In 11.7% of the patients, the patient was sent home from the ED and not admitted to the hospital, and thus deemed suitable for non-conveyance. Of these patients, 13.3% received antibiotics at the ED. A NEWS of zero had a sensitivity of 8.9% for identifying non-admitted patients. The PPV was 32.0%. Adding WBC, CRP or lactate to the NEWS zero further reduced the sensitivity, but the PPV still remained < 80% (Table 4).

**Table 4:** Accuracy of NEWS, CRP, WBC and Lactate for identifaction of patients not admitted to hospital

	Sensitivity (CI 95%)	Specificity (CI 95%)	PPV (CI 95%)	NPV (CI 95%)	Odds ratio (CI 95%)	Adjusted odds ratio* CI 95%)
**0 points NEWS**	8.9 (6.8–11.4)	97.5 (97.0-97.9)	32.0 (25.7–39.0)	89.0 (88.7–89.2)	3.8 (2.7–5.3)	3.5 (2.4-5.0)
**0 points NEWS and WBC 3.5-8.8 × 10** ^**9**^ **/L**	5.1 (3.5–7.1)	99.2 (98.9–99.4)	45.1 (34.1–56.5)	88.8 (88.6–88.9)	6.4 (4.0-10.4)	2.5 (1.9–3.1)
**0 points NEWS and CRP < 10 mg/L**	2.7 (1.6–4.3)	100.0 (99.8–100.0)	77.3 (55.7–90.2)	88.6 (88.4–88.7)	26.4 (9.7–71.7)	3.6 (2.9–4.5)
**0 points NEWS and Lactate 0**,**5 − 2**,**2 mmol/L**	7.6 (5.6–10.0)	97.9 (97.5–98.3)	32.7 (25.8–40.4)	88.9 (88.7–89.1)	3.9 (2.7–5.5)	2.2 (1.8–2.6)
**0 points NEWS and one of the following: WBC 3.5-8.8 × 10**^**9**^**/L**,** CRP < 10 mg/L and Lactate 0**,**5 − 2**,**2 mmol/L**	8.2 (6.2–10.7)	97.8 (97.4–98.2)	33.3 (26.6–40.8)	89.0 (88.7–89.2)	4.0 (2.9–5.7)	-

CI = Confidence Interval, PPV = Positive predictive value, NPV = Negative predictive value, WBC = White Blood Cell Count, CRP = C-Reactive Protein, NEWS = National Early Warning Score

*Multivariate logistic regression including 0 points NEWS, WBC 3.5-8.8 × 109/L, CRP < 10 mg/L and Lactate 0,5 − 2,2 mmol/L

A NEWS of zero, WBC, CRP and lactate all turned out to a have statistically significant odds ratio for the identification of non-admitted patients (*p* < 0.001). A NEWS of zero and a CRP < 10 mg/l were the strongest predictors (Table 4).

## Discussion

The current study is one of the larger studies evaluating the accuracy of sepsis screening tools in the EMS setting, including 5,405 patients for whom infection was suspected by EMS personnel. A total of 9.8% of these patients were diagnosed with sepsis during their hospital stay. This is in line with the incidence reported by Guerra et al. [22]. The screening tools examined showed variable accuracy, with none demonstrating both satisfactory sensitivity and specificity. Adding blood tests such as CRP, WBC, and lactate did not significantly improve sepsis identification. The blood tests included all had a predictive value for identifying both patients with sepsis and patients not admitted to the hospital. However, blood tests added limited prognostic information for the identification of sepsis. Previous studies by Olander et al. [35] and Wallgren et al. [36] reported that lactate has small predictive value for sepsis identification. Boland et al. [19] showed that lactate did not significantly improve sepsis identification. This is also true for Jousi et al. [24], who reported that neither lactate nor CRP added any valuable information when assessing EMS patients with nonspecific complaints. These results highlight the inherent challenges in accurately identifying sepsis in the prehospital setting.

The results of this study, compared with a large published prehospital validation study by Lane et al. [17], show a difference in the c-statistics for the screening tools that are common between the studies. In general, our study reported poorer specificity and PPV, while the NPV was greater. For sensitivity, there was no clear pattern. Several factors may contribute to these variations. Firstly, Lane et al. [17] included EMS patients who had an infection diagnosed in the ED, while we included patients who had suspected infection as assessed by EMS personnel. This difference in patient selection criteria could account for some of the disparities observed. Secondly, they excluded patients discharged from the ED. This was not the case in our study. Thirdly, they defined sepsis as being.*“diagnosed with infection in the emergency department and |…| found to have organ dysfunction characteristic of sepsis. Organ dysfunction was identified from diagnostic codes or altered vital signs consistent with organ dysfunction…”*. This results in twice as high an incidence of sepsis. Finally, our study was conducted in the COVID-19 era, and approximately 10% of the cases included were diagnosed with COVID-19. The screening tools examined were developed before the COVID-19 outbreak in 2020, and they also included their patients before 2020. This contextual difference may influence the comparability and interpretation of results between the studies. Unfortunately, c-statistics were not reported for the NEWS by Lane et al. [17]. However, the NEWS, when used as a continuous numbered score, shows good discrimination and calibration, described as a high probability of sepsis when it has a high score and vice versa. This is supported by our findings in which the NEWS achieved relatively good overall c-statistics. This finding is also in line with the findings of Wallgren et al. [16].

The results of this study showed a mean time reduction of 64 min from EMS arrival to antibiotic administration, when comparing sepsis patients given the highest priority according to RETTS triage with sepsis patients with lower priority. This indicates that high EMS triage priority has a significant effect on ED patient care and that early sepsis identification promotes substantial time gain. This finding is also supported by previous studies [6, 8] and has the potential to improve patient outcomes [6, 7, 9]. However, promptly administering antibiotics based on EMS sepsis submission without a prior confirmed diagnosis can increase the likelihood of developing multidrug resistance [37]. Such misuse can thus jeopardise individual health and burden national healthcare systems economically [38].

It could be argued that the RETTS triage level is more indicative of acute severity than the diagnosis of sepsis, given the substantial difference in time to antibiotic administration for different triage levels. However, ad-hoc analyses shows that both seven- and thirty-day mortality rates are more strongly associated with diagnosis of sepsis than with red triage level, indicating that this is not the case.

The study also highlights the complexity of identifying EMS patients suitable for non-conveyance, given the high admission rates and mortality in this cohort compared with other EMS patient groups studied in the Swedish EMS setting [39–41]. This is especially the case since some non-admitted patients also received antibiotics at the ED. If these patients had been non-conveyed, they would have needed to be referred to a primary care centre or to have antibiotics prescribed on scene. Even though CRP, WBC, and lactate showed some predictive value for identifying patients not requiring hospital admission, their practical benefit remains limited due to the small number of such patients in the EMS infection-suspected group. This underlines the difficulty in safely implementing non-conveyance decisions for patients with suspected infections [42]. Future research should focus on assessing the accuracy of EMS evaluations in non-conveyed patients and exploring the potential of integrating screening tools or blood tests to improve decision-making in this high-risk group.

### Strengths and limitations

The study’s inclusion of a large, unselected patient cohort with suspected infections in the EMS setting enhances the generalisability of the findings to clinical care. This approach provides valuable insights into real-world EMS operations in which the diagnosis of infection is made without the benefit of ED confirmation. The study is notable for being one of the first to evaluate the accuracy of sepsis screening tools in the EMS setting during the COVID-19 era, making the results directly relevant to current clinical practice. Additionally, using EMS-based data reflects the immediate conditions faced by EMS personnel, providing practical insights into prehospital care.

One limitation is that the study included only patients suspected of infection by EMS personnel, potentially missing those with atypical presentations like dyspnoea or non-specific symptoms who may also have infections. The application of sepsis screening tools to these broader patient groups remains uncertain and warrants further study. This study relied on sepsis diagnoses based on ICD codes, which may underestimate the true incidence as coding practices vary and may not capture all cases of sepsis. Additionally, the use of ED blood test results as surrogates for prehospital testing introduces variability, as results may differ due to timing and equipment differences. Using ED blood sample results as surrogate measures for EMS equivalents is deemed an appropriate method for examining their potential value in the EMS setting since introducing EMS POCT analyses is costly and demanding.

## Conclusions

Identifying sepsis in EMS settings remains challenging, with existing screening tools offering limited accuracy. CRP, WBC, and lactate blood tests add minimal predictive value in distinguishing sepsis or determining non-conveyance eligibility. The challenge of identifying EMS patients with infection who do not require hospital admission underlines the need for improved diagnostic strategies.

## Electronic supplementary material

Below is the link to the electronic supplementary material.

Supplementary Material 1 Additional file 1 List and frequency of ICD-10 codes at emergency department

Supplementary Material 2 Additional file 2 List and frequency of ICD-10 codes at discharge after hospital admission

Supplementary Material 3 Additional file 3 Bar chart of screening tool accuracy

Supplementary Material 4 Additional file 4 Forest plot of screening tool accuracy

## Data Availability

The datasets generated and analysed during the current study are not publicly available due to the integrity of patient privacy but are available from the corresponding author upon reasonable request and if approved by the Swedish ethical review authority.

## Abbreviations

BAS: Blodtryck andningsfrekvens saturation (Swedish for Blood pressure Breathing Rate Saturation)
CIP: Critical illness prediction
CRP: C-Reactive protein
ED: Emergency department
EMS: Emergency medical services
EMDC: Emergency medical dispatch centre
ESS: Emergency signs and symptoms
HR: Heart rate
ICD: International statistical classification of diseases and related health problems
ICU: Intensive care unit
NEWS: National early warning score
NPV: Negative predictive value
POCT: Point of care testing
PPV: Positive predictive value
PRESS: Prehospital severe sepsis score
qSOFA: Quick sepsis-related organ failure assessment
RR: Respiratory rate
RETTS: Rapid emergency triage and treatment system
SIRS: Systemic inflammatory response syndrome
WBC: White blood cell
