# Supplementary material for: The performance of screening tools and use of blood analyses in prehospital identification of sepsis patients and patients suitable for non-conveyance - an observational study
Source: BMC Emerg Med. 2024 Oct 8;24:180. doi: 10.1186/s12873-024-01098-4 (PMC11462654; doi:10.1186/s12873-024-01098-4)
Supplement: Supplementary file 1 — Supplementary Material 1 Additional file 1 List and frequency of ICD-10 codes at emergency department [file 12873_2024_1098_MOESM1_ESM.pdf]

Additional file 1- List and frequency of ICD-10 codes at emergency department

ICD-diagnoses are translated from Swedish using Chat GPT, version 3.5.

**ICD-10 code**	**n**	**%**
R509 - Fever, unspecified	1417	26
B999 - Other infectious disease	617	11
U071 - COVID-19, virus identified	424	8
N390 - Urinary tract infection, site not specified	352	6
J189 - Pneumonia, unspecified	309	6
R060 - Dyspnea	195	4
A419 - Sepsis, unspecified	178	3
A469 - Erysipelas	153	3
N109 - Acute tubulo-interstitial nephritis	142	3
R539 - Malaise and fatigue	141	3
T835 - Infection and inflammatory reaction due to internal prosthetic devices, implants and grafts in urinary system	105	2
J101 - Influenza with other respiratory manifestations, seasonal influenza virus identified	68	1
J159 - Pneumonia, unspecified	65	1
R104 - Abdominal pain unspecified	41	1
R104 - Other and unspecified abdominal pain	41	1
A099 - Gastroenteritis and colitis of unspecified origin	40	1
T814 - Infection following a procedure, not elsewhere classified	40	1
U999 - Diagnosis code not available	38	1
B349 - Measles without complications	36	1
A499 - Bacterial infection, unspecified	32	1
J069 - Acute upper respiratory infection, unspecified	29	1
L089 - Local infection of skin and subcutaneous tissue, unspecified	29	1
R508 - Other specified fever	29	1
R119 - Nausea	28	1
R119 - Nausea and vomiting	28	1
R119 - Vomiting	28	1
J441 - Chronic obstructive pulmonary disease with (acute) exacerbation, unspecified	25	0
L979 - Disorder of skin and subcutaneous tissue, unspecified	25	0
M796 - Other soft tissue disorders related to use, overuse and pressure, unspecified	22	0
R410 - Unspecified delirium	20	0
J690 - Pneumonitis due to food and vomit	18	0
L899 - Disorder of skin and subcutaneous tissue, unspecified	16	0
I509 - Heart failure, unspecified	14	0
J987 - Respiratory disorders in diseases classified elsewhere	14	0
K830 - Acute pancreatitis	14	0
R059 - Cough	14	0
N179 - Renal tubulo-interstitial disease, unspecified	13	0

R298 - Other and unspecified symptoms and signs involving the nervous and musculoskeletal systems	12	0
J108 - Influenza due to other identified influenza virus with other manifestations	11	0
K859 - Appendicitis, unspecified	11	0
R074 - Chest pain, unspecified	11	0
R400 - Somnolence	11	0
E871 - Adverse effect of antineoplastic and immunosuppressive drugs, initial encounter	10	0
I702 - Atherosclerosis of aorta	10	0
K810 - Acute cholecystitis with obstruction	10	0
L031 - Cellulitis of other parts of limb	10	0
T793 - Post-traumatic wound infection, not elsewhere classified	10	0
D649 - Anemia, unspecified	9	0
E869 - Unspecified adverse effect of antineoplastic and immunosuppressive drugs	9	0
B029 - Zoster without complications	8	0
R559 - Fainting and collapse	8	0
E116 - Type 1 diabetes mellitus, with multiple complications	7	0
E876 - Adverse effect of other drugs, medicaments and biological substances, subsequent encounter	7	0
K591 - Diverticulitis of colon without perforation or abscess	7	0
M549 - Dorsalgia, unspecified	7	0
R519 - Headache	7	0
U072 - COVID-19, virus not identified	7	0
A047 - Enteropathy due to other bacterial foodborne intoxications	6	0
I489 - Atrial fibrillation and flutter, unspecified	6	0
J030 - Acute tonsillitis due to other specified organisms	6	0
J039 - Acute tonsillitis, unspecified	6	0
K922 - Follicular cyst of skin and subcutaneous tissue	6	0
M000 - Pyogenic arthritis	6	0
M702 - Other soft tissue disorders related to use, overuse and pressure, unspecified	6	0
N136 - Pyonephrosis	6	0
R068 - Other and unspecified abnormalities of breathing	6	0
Z038 - Observation for suspected diseases and conditions ruled out	6	0
Z038 - Observation for suspected infectious disease (bacterial, viral) ruled out	6	0
Z038 - Observation for suspected other specified disease ruled out	6	0
L984 - Other specified local infections of skin and subcutaneous tissue	5	0
M139 - Monoarthritis, unspecified	5	0
N459 - Inflammatory disease of male pelvic organs, unspecified	5	0
S065 - Traumatic subdural hemorrhage	5	0
T845 - Infection and inflammatory reaction due to internal joint prosthesis of hip and thigh	5	0
T845 - Infection and inflammatory reaction due to internal joint prosthesis of knee and lower leg	5	0
T845 - Infection and inflammatory reaction due to internal joint prosthesis of shoulder and upper arm	5	0
A081 - Rotaviral enteritis	4	0
E119 - Type 1 diabetes mellitus, without complications	4	0

E870 - Adverse effect of antineoplastic and immunosuppressive drugs, unspecified	4	0
I959 - Hypertension, unspecified	4	0
J100 - Influenza due to other identified influenza virus with pneumonia	4	0
J111 - Influenza with other respiratory manifestations, virus not identified	4	0
J440 - Chronic obstructive pulmonary disease with acute lower respiratory infection	4	0
L024 - Cutaneous abscess, furuncle and carbuncle of limb	4	0
L033 - Cellulitis of face	4	0
L088 - Other specified local infections of skin and subcutaneous tissue	4	0
M100 - Idiopathic gout, unspecified	4	0
N129 - Tubulo-interstitial nephritis, not specified as acute or chronic, unspecified	4	0
R296 - Predisposition to falling, not elsewhere classified	4	0
R401 - Stupor	4	0
R429 - Dizziness and giddiness	4	0
R529 - Pain, unspecified	4	0
T509 - Other and unspecified drugs, medicaments and biological substances	4	0
T857 - Infection and inflammatory reaction due to other internal prosthetic devices, implants and grafts	4	0
C349 - Malignant neoplasm of unspecified part of unspecified breast	3	0
C349 - Malignant neoplasm of unspecified part of unspecified female breast	3	0
C349 - Malignant neoplasm of unspecified part of unspecified male breast	3	0
C795 - Secondary malignant neoplasm of other and unspecified parts of nervous system	3	0
D709 - Coagulation defect, unspecified	3	0
E101 - Non-insulin-dependent diabetes mellitus, with ketoacidosis	3	0
G459 - Transient cerebral ischemic attack, unspecified	3	0
I269 - Pulmonary embolism without acute cor pulmonale	3	0
J019 - Acute sinusitis, unspecified	3	0
J099 - Acute upper respiratory infection, unspecified	3	0
J118 - Influenza with other manifestations, virus not identified	3	0
J121 - Avian influenza, due to identified avian influenza virus	3	0
J149 - Pneumonia, unspecified organism	3	0
J369 - Chronic upper respiratory infection, unspecified	3	0
K529 - Unspecified disease of anus and rectum	3	0
K819 - Cholecystitis, unspecified	3	0
M869 - Chronic osteomyelitis, unspecified	3	0
N300 - Cyst of kidney, acquired	3	0
R189 - Ascites	3	0
R339 - Retention of urine	3	0
S720 - Intracapsular fracture of femoral neck, closed	3	0
S800 - Contusion of knee	3	0
T784 - Allergy, unspecified	3	0
T846 - Infection and inflammatory reaction due to internal fixation device, hip and thigh	3	0
T846 - Infection and inflammatory reaction due to internal fixation device, knee and lower leg	3	0
T846 - Infection and inflammatory reaction due to internal fixation device, unspecified site	3	0

A049 - Bacterial intestinal infection, unspecified	2	0
B279 - Mumps without complication	2	0
C259 - Malignant neoplasm of pancreas, unspecified	2	0
C619 - Malignant neoplasm of endometrium, unspecified	2	0
C833 - Malignant neoplasm of paraganglia	2	0
C911 - Chronic lymphocytic leukemia of B-cell type	2	0
D630 - Anemia due to antineoplastic chemotherapy	2	0
E109 - Non-insulin-dependent diabetes mellitus, without complications	2	0
E111 - Type 1 diabetes mellitus, with coma	2	0
E875 - Adverse effect of other drugs, medicaments and biological substances, initial encounter	2	0
G009 - Bacterial meningitis, unspecified	2	0
I620 - Subarachnoid hemorrhage from middle cerebral artery	2	0
J029 - Acute sinusitis, unspecified	2	0
J139 - Unspecified acute lower respiratory infection	2	0
J205 - Acute bronchitis due to unspecified organism	2	0
J229 - Acute bronchiolitis due to other specified organisms	2	0
J909 - Respiratory abnormality, unspecified	2	0
K567 - Diverticulitis of small intestine without perforation or abscess without bleeding	2	0
K573 - Unspecified diverticulitis of large intestine	2	0
K590 - Diverticulitis of colon without perforation or abscess	2	0
K610 - Anal fissure, unspecified	2	0
K650 - Perianal venous thrombosis	2	0
K768 - Other specified diseases of anus and rectum	2	0
K831 - Chronic pancreatitis	2	0
K920 - Infectious folliculitis	2	0
K921 - Noninfective folliculitis	2	0
L010 - Impetigo [any organism]	2	0
L030 - Cellulitis of finger and toe	2	0
M109 - Gout, unspecified	2	0
M255 - Pain in unspecified joint	2	0
M464 - Vertebrogenic disorders of back, not elsewhere classified	2	0
M542 - Cervicalgia	2	0
M600 - Deformity of finger(s), acquired	2	0
M704 - Other soft tissue disorders related to use, overuse and pressure, shoulder region	2	0
M791 - Myalgia	2	0
N200 - Calculus of kidney	2	0
N201 - Calculus of ureter	2	0
N239 - Calculus of lower urinary tract, unspecified	2	0
N309 - Unspecified hydronephrosis	2	0
R000 - Tachycardia, unspecified	2	0
R073 - Other chest pain	2	0
R093 - Abnormal sputum	2	0
R103 - Pain localized to other parts of lower abdomen	2	0

R179 - Hyperbilirubinemia without mention of jaundice, not elsewhere classified	2	0
R221 - Localized swelling, mass, or lump in neck	2	0
R319 - Unspecified hematuria (blood in urine)	2	0
R402 - Coma, unspecified	2	0
R568 - Seizures, unspecified	2	0
R799 - Abnormal finding of blood chemistry, unspecified	2	0
S009 - Superficial injury of head, unspecified	2	0
S300 - Contusion of lower back and pelvis	2	0
S320 - Closed fracture of lumbar spine	2	0
T141 - Bite of other specified venomous animal, unspecified	2	0
T802 - Infection following infusion, transfusion and therapeutic injection	2	0
T810 - Bleeding and hematoma complicating a procedure, not elsewhere classified	2	0
T827 - Infection and inflammatory reaction due to other cardiac and vascular devices, implants and grafts	2	0
T831 - Mechanical complication of other internal prosthetic devices, implants and grafts in urinary tract	2	0
T874 - Infection of amputation stump	2	0
T874 - Infection of amputation stump, hip and thigh	2	0
T874 - Infection of amputation stump, knee and lower leg	2	0
A020 - Enterotoxigenic Escherichia coli infection	1	0
A043 - Enterohemorrhagic Escherichia coli infection	1	0
A045 - Other Escherichia coli intestinal infections	1	0
A084 - Other specified viral intestinal infections	1	0
A090 - Other and unspecified gastroenteritis and colitis of infectious origin	1	0
A403 - Sepsis due to streptococcus, other and unspecified	1	0
A491 - Streptococcal infection, unspecified	1	0
A498 - Other bacterial diseases, not elsewhere classified	1	0
B270 - Mumps meningitis	1	0
B270 - Mumps with other complications	1	0
B342 - Measles keratoconjunctivitis	1	0
B370 - Candidal stomatitis	1	0
C021 - Malignant neoplasm of rectosigmoid junction	1	0
C169 - Malignant neoplasm of stomach, unspecified part	1	0
C209 - Malignant neoplasm of colon, unspecified	1	0
C250 - Malignant neoplasm of pancreas	1	0
C509 - Malignant neoplasm of unspecified part of the uterus	1	0
C569 - Malignant neoplasm of unspecified part of the colon	1	0
C780 - Secondary malignant neoplasm of lung	1	0
C787 - Secondary malignant neoplasm of liver and intrahepatic bile duct	1	0
C793 - Secondary malignant neoplasm of brain and cerebral meninges	1	0
C819 - Kaposi's sarcoma, unspecified	1	0
C830 - Malignant neoplasm of adrenal gland	1	0
C950 - Leukemia of unspecified cell type	1	0
D462 - Refractory anemia with excess of blasts	1	0
D509 - Neoplasm of uncertain behavior of digestive organ, unspecified	1	0

D699 - Hemorrhagic condition, unspecified	1	0
E106 - Non-insulin-dependent diabetes mellitus, with other specified complications	1	0
E110 - Type 1 diabetes mellitus, with ketoacidosis	1	0
E115 - Type 1 diabetes mellitus, with other specified complications	1	0
E271 - Primary adrenocortical insufficiency	1	0
E272 - Other adrenocortical insufficiency	1	0
E835 - Therapeutic use of antineoplastic agents causing adverse effects in therapeutic use	1	0
E872 - Adverse effect of antineoplastic and immunosuppressive drugs, subsequent encounter	1	0
F103 - Mental and behavioral disorders due to use of alcohol, withdrawal state with delirium	1	0
G039 - Meningitis, unspecified	1	0
G122 - Cervical spinal cord injury without evidence of spinal bone injury	1	0
G500 - Essential tremor	1	0
G728 - Other specified disorders of central nervous system	1	0
H651 - Labyrinthine fistula, congenital	1	0
H660 - Otitis media, unspecified	1	0
H669 - Otitis media, unspecified, bilateral	1	0
I313 - Pericarditis, unspecified	1	0
I499 - Other forms of cardiac arrhythmia	1	0
I609 - Intracranial hemorrhage, unspecified	1	0
I619 - Intracerebral hemorrhage, unspecified	1	0
I802 - Phlebitis and thrombophlebitis of superficial vessels of unspecified lower extremity	1	0
I950 - Hypertension secondary to renal disease	1	0
J028 - Acute sinusitis, other	1	0
J154 - Pneumonia due to other specified bacteria	1	0
J156 - Pneumonia due to other infectious organisms	1	0
J180 - Bronchopneumonia, unspecified	1	0
J209 - Acute bronchitis, unspecified	1	0
J210 - Acute bronchiolitis due to respiratory syncytial virus	1	0
J399 - Unspecified pharyngitis	1	0
J449 - Chronic obstructive pulmonary disease, unspecified	1	0
J459 - Chronic bronchitis, unspecified	1	0
J919 - Respiratory condition, unspecified	1	0
K047 - Chronic pancreatitis	1	0
K049 - Chronic pancreatitis, unspecified	1	0
K089 - Other specified disorders of pancreas	1	0
K121 - Acute duodenal ulcer with hemorrhage	1	0
K122 - Acute duodenal ulcer with perforation	1	0
K137 - Unspecified duodenal ulcer as acute or chronic, without hemorrhage or perforation	1	0
K309 - Duodenitis, unspecified	1	0
K353 - Fissure and fistula of anal canal	1	0
K358 - Other specified diseases of anus and rectum	1	0
K579 - Diverticulitis of colon without perforation or abscess	1	0

K611 - Anal fissure, chronic	1	0
K625 - Anal abscess	1	0
K661 - Ulcer of anal canal	1	0
K729 - Diseases of anus and rectum, unspecified	1	0
K801 - Acute cholecystitis	1	0
K802 - Calculus of gallbladder with acute cholecystitis	1	0
K803 - Calculus of gallbladder without cholecystitis	1	0
K851 - Acute appendicitis	1	0
L020 - Cutaneous abscess, furuncle and carbuncle	1	0
L023 - Cutaneous abscess, furuncle and carbuncle of buttock	1	0
L028 - Cutaneous abscess, furuncle and carbuncle of other sites	1	0
L038 - Cellulitis of other specified sites	1	0
L039 - Cellulitis, unspecified	1	0
L042 - Cutaneous abscess, furuncle and carbuncle of face	1	0
L050 - Pilonidal cyst with abscess	1	0
L059 - Pilonidal cyst without abscess	1	0
L739 - Lichenoid drug reaction, unspecified	1	0
L959 - Reaction to unspecified agent, unspecified	1	0
L989 - Disorder of skin and subcutaneous tissue, unspecified	1	0
M009 - Local infection of unspecified site	1	0
M118 - Other juvenile arthritis	1	0
M254 - Effusion, unspecified	1	0
M316 - Recurrent dislocation of joint	1	0
M353 - Dislocation of joint, unspecified	1	0
M485 - Other current conditions in the musculoskeletal system and connective tissue, specified	1	0
M544 - Lumbago with sciatica	1	0
M703 - Calcific tendinitis	1	0
M861 - Subacute hematogenous osteomyelitis	1	0
M870 - Osteonecrosis due to previous trauma, unspecified	1	0
N110 - Chronic tubulo-interstitial nephritis in diseases classified elsewhere	1	0
N111 - Chronic tubulo-interstitial nephritis, not specified as acute or chronic	1	0
N185 - Chronic kidney disease, stage 5	1	0
N189 - Chronic kidney disease, unspecified	1	0
N433 - Vesicoureteral-reflux with reflux nephropathy	1	0
N489 - Inflammatory disorder of the male genital organs, unspecified	1	0
N508 - Other specified inflammatory diseases of female pelvic organs	1	0
N509 - Inflammatory disease of female pelvic organs, unspecified	1	0
O860 - Other specified complications of labor and delivery, delivered, with or without mention of antepartum condition	1	0
O995 - Endocrine, nutritional and metabolic diseases complicating pregnancy, childbirth, and the puerperium	1	0
R040 - Epistaxis	1	0
R071 - Chest pain on breathing	1	0
R091 - Pleuritic pain	1	0
R101 - Pain localized to upper abdomen	1	0

R139 - Difficulty in swallowing	1	0
R170 - Hyperbilirubinemia with mention of jaundice, not elsewhere classified	1	0
R202 - Paresthesia	1	0
R219 - Unspecified rash	1	0
R220 - Localized swelling, mass, or lump in head	1	0
R222 - Localized swelling, mass, or lump on trunk	1	0
R238 - Other specified skin changes	1	0
R418 - Other and unspecified cognitive disturbances	1	0
R441 - Visual hallucinations	1	0
R502 - Drug-induced fever	1	0
R579 - Shock, unspecified	1	0
R680 - Hypothermia not associated with low environmental temperature	1	0
S202 - Contusion of chest wall	1	0
S422 - Fracture of upper end of humerus, open	1	0
S500 - Contusion of elbow	1	0
S519 - Open wound of forearm, unspecified	1	0
S819 - Open wound of lower leg, unspecified	1	0
S836 - Sprain of other and unspecified parts of knee	1	0
T140 - Hematoma, unspecified	1	0
T670 - Heatstroke and sunstroke	1	0
T689 - Hypothermia	1	0
T783 - Angioneurotic edema	1	0
T796 - Traumatic ischemia of muscle	1	0
T830 - Mechanical complication of other vascular grafts	1	0
T834 - Mechanical complication of other internal orthopedic devices, implants and grafts	1	0
T836 - Infection and inflammatory reaction due to internal prosthetic devices, implants and grafts in genital organs	1	0
T838 - Other specified complications of internal prosthetic devices, implants and grafts in urogenital system	1	0
T840 - Mechanical complication of internal joint prosthesis of hip	1	0
T847 - Infection and inflammatory reaction due to other internal orthopedic prosthetic devices, implants and grafts, ankle and foot	1	0
T889 - Complications of surgical and medical care, unspecified	1	0
U099 - Post-COVID-19 condition, unspecified	1	0
Z340 - Supervision of normal first pregnancy	1	0
Z452 - Adjustment and management of vascular access device	1	0
Z711 - Person encountering health services for other specified examination and observation	1	0
Z719 - Counseling, unspecified	1	0
Z742 - Need for assistance at home and absence of other household member able to render care	1	0
Z768 - Other specified contact with health services	1	0
