# Supplementary material for: The performance of screening tools and use of blood analyses in prehospital identification of sepsis patients and patients suitable for non-conveyance - an observational study
Source: BMC Emerg Med. 2024 Oct 8;24:180. doi: 10.1186/s12873-024-01098-4 (PMC11462654; doi:10.1186/s12873-024-01098-4)
Supplement: Supplementary file 2 — Supplementary Material 2 Additional file 2 List and frequency of ICD-10 codes at discharge after hospital admission [file 12873_2024_1098_MOESM2_ESM.pdf]

Additional file 2- List and frequency of ICD-10 codes at discharge after hospital admission

ICD-diagnoses are translated from Swedish using Chat GPT, version 3.5.

**ICD-10 code**	**n**	**%**
J159 - Bacterial pneumonia, unspecified	489	10
U071 - COVID-19, virus identified	415	9
N109 - Acute tubulo-interstitial nephritis	394	8
N390 - Urinary tract infection, site not specified	379	8
T835 - Infection and inflammatory reaction due to internal prosthetic devices, implants and grafts in urinary system	248	5
A469 - Erysipelas	224	5
B999 - Other infectious disease	176	4
R509 - Fever, unspecified	156	3
J189 - Pneumonia, unspecified	101	2
J690 - Pneumonitis due to food and vomit	82	2
T814 - Infection following a procedure, not elsewhere classified	71	1
A499 - Bacterial infection, unspecified	65	1
J101 - Influenza with other respiratory manifestations, seasonal influenza virus identified	51	1
A419 - Sepsis, unspecified	50	1
A415 - Sepsis due to other Gram-negative organisms	48	1
J441 - Chronic obstructive pulmonary disease with (acute) exacerbation, unspecified	42	1
R508 - Other specified fever	41	1
A410 - Septicemia due to Staphylococcus aureus	39	1
L979 - Disorder of skin and subcutaneous tissue, unspecified	39	1
T827 - Infection and inflammatory reaction due to other cardiac and vascular devices, implants and grafts	39	1
B349 - Measles without complications	36	1
J069 - Acute upper respiratory infection, unspecified	36	1
A047 - Enteropathy due to other bacterial foodborne intoxications	34	1
J149 - Pneumonia, unspecified organism	31	1
L031 - Cellulitis of other parts of limb	30	1
N136 - Pyonephrosis	30	1
J139 - Unspecified acute lower respiratory infection	28	1
A498 - Other bacterial diseases, not elsewhere classified	27	1
K830 - Acute pancreatitis	27	1
I509 - Heart failure, unspecified	24	0
I702 - Atherosclerosis of aorta	24	0
L899 - Disorder of skin and subcutaneous tissue, unspecified	23	0
J987 - Respiratory disorders in diseases classified elsewhere	20	0
K800 - Chronic cholecystitis	18	0
N179 - Renal tubulo-interstitial disease, unspecified	18	0
A490 - Streptococcal sore throat	17	0
A491 - Streptococcal infection, unspecified	17	0

K803 - Calculus of gallbladder without cholecystitis	17	0
I269 - Pulmonary embolism without acute cor pulmonale	16	0
R539 - Malaise and fatigue	16	0
T857 - Infection and inflammatory reaction due to other internal prosthetic devices, implants and grafts	16	0
T845 - Infection and inflammatory reaction due to internal joint prosthesis of hip and thigh	15	0
T845 - Infection and inflammatory reaction due to internal joint prosthesis of knee and lower leg	15	0
T845 - Infection and inflammatory reaction due to internal joint prosthesis of shoulder and upper arm	15	0
J100 - Influenza due to other identified influenza virus with pneumonia	14	0
A099 - Gastroenteritis and colitis of unspecified origin	13	0
M000 - Pyogenic arthritis	13	0
N309 - Unspecified hydronephrosis	13	0
A081 - Rotaviral enteritis	12	0
C349 - Malignant neoplasm of unspecified part of unspecified breast	11	0
C349 - Malignant neoplasm of unspecified part of unspecified female breast	11	0
C349 - Malignant neoplasm of unspecified part of unspecified male breast	11	0
C619 - Malignant neoplasm of endometrium, unspecified	11	0
K529 - Unspecified disease of anus and rectum	11	0
M100 - Idiopathic gout, unspecified	11	0
B029 - Zoster without complications	10	0
I330 - Acute infective endocarditis	10	0
J121 - Avian influenza, due to identified avian influenza virus	10	0
J158 - Pneumonia, unspecified organism	10	0
J209 - Acute bronchitis, unspecified	10	0
M109 - Gout, unspecified	10	0
I501 - Left ventricular failure	9	0
I639 - Cerebral infarction, unspecified	9	0
K851 - Acute appendicitis	9	0
M796 - Other soft tissue disorders related to use, overuse and pressure, unspecified	9	0
R060 - Dyspnea	9	0
R410 - Unspecified delirium	9	0
A400 - Sepsis due to streptococcus, group A	8	0
E869 - Unspecified adverse effect of antineoplastic and immunosuppressive drugs	8	0
E876 - Adverse effect of other drugs, medicaments and biological substances, subsequent encounter	8	0
J152 - Pneumonia due to other streptococci	8	0
J156 - Pneumonia due to other infectious organisms	8	0
K650 - Perianal venous thrombosis	8	0
K810 - Acute cholecystitis with obstruction	8	0
L033 - Cellulitis of face	8	0
A045 - Other Escherichia coli intestinal infections	7	0
C250 - Malignant neoplasm of pancreas	7	0
D649 - Anemia, unspecified	7	0
J151 - Pneumonia due to Streptococcus pneumoniae	7	0

M869 - Chronic osteomyelitis, unspecified	7	0
R502 - Drug-induced fever	7	0
S065 - Traumatic subdural hemorrhage	7	0
S720 - Intracapsular fracture of femoral neck, closed	7	0
A401 - Sepsis due to streptococcus, group B	6	0
A411 - Septicemia due to other specified staphylococcus	6	0
B485 - Enterovirus as the cause of diseases classified elsewhere	6	0
C833 - Malignant neoplasm of paraganglia	6	0
I489 - Atrial fibrillation and flutter, unspecified	6	0
J108 - Influenza due to other identified influenza virus with other manifestations	6	0
J229 - Acute bronchiolitis due to other specified organisms	6	0
J869 - Respiratory failure, unspecified	6	0
K572 - Diverticulitis of large intestine with perforation or abscess with bleeding	6	0
K819 - Cholecystitis, unspecified	6	0
K859 - Appendicitis, unspecified	6	0
K922 - Follicular cyst of skin and subcutaneous tissue	6	0
M353 - Dislocation of joint, unspecified	6	0
M702 - Other soft tissue disorders related to use, overuse and pressure, unspecified	6	0
M861 - Subacute hematogenous osteomyelitis	6	0
N300 - Cyst of kidney, acquired	6	0
A020 - Enterotoxigenic Escherichia coli infection	5	0
C259 - Malignant neoplasm of pancreas, unspecified	5	0
C920 - Acute myeloid leukemia with t(8;21)(q22;q22)	5	0
E835 - Therapeutic use of antineoplastic agents causing adverse effects in therapeutic use	5	0
I500 - Congestive heart failure	5	0
K573 - Unspecified diverticulitis of large intestine	5	0
L089 - Local infection of skin and subcutaneous tissue, unspecified	5	0
N201 - Calculus of ureter	5	0
R319 - Unspecified hematuria (blood in urine)	5	0
R339 - Retention of urine	5	0
T509 - Other and unspecified drugs, medicaments and biological substances	5	0
T818 - Insufficiency of anastomosis	5	0
T818 - Other specified complications of procedures, not elsewhere classified	5	0
T838 - Other specified complications of internal prosthetic devices, implants and grafts in urogenital system	5	0
T874 - Infection of amputation stump	5	0
T874 - Infection of amputation stump, hip and thigh	5	0
T874 - Infection of amputation stump, knee and lower leg	5	0
A403 - Sepsis due to streptococcus, other and unspecified	4	0
A414 - Other gram-negative sepsis	4	0
A418 - Other specified sepsis	4	0
B270 - Mumps meningitis	4	0
B270 - Mumps with other complications	4	0
C793 - Secondary malignant neoplasm of brain and cerebral meninges	4	0

C795 - Secondary malignant neoplasm of other and unspecified parts of nervous system	4	0
D509 - Neoplasm of uncertain behavior of digestive organ, unspecified	4	0
E116 - Type 1 diabetes mellitus, with multiple complications	4	0
I620 - Subarachnoid hemorrhage from middle cerebral artery	4	0
I959 - Hypertension, unspecified	4	0
J030 - Acute tonsillitis due to other specified organisms	4	0
J150 - Pneumonia due to Haemophilus influenzae	4	0
J154 - Pneumonia due to other specified bacteria	4	0
J369 - Chronic upper respiratory infection, unspecified	4	0
J440 - Chronic obstructive pulmonary disease with acute lower respiratory infection	4	0
J909 - Respiratory abnormality, unspecified	4	0
K567 - Diverticulitis of small intestine without perforation or abscess without bleeding	4	0
K590 - Diverticulitis of colon without perforation or abscess	4	0
K610 - Anal fissure, unspecified	4	0
K801 - Acute cholecystitis	4	0
K805 - Acute cholecystitis with chronic cholecystitis	4	0
L030 - Cellulitis of finger and toe	4	0
M118 - Other juvenile arthritis	4	0
N459 - Inflammatory disease of male pelvic organs, unspecified	4	0
R119 - Nausea	4	0
R119 - Nausea and vomiting	4	0
R119 - Vomiting	4	0
R429 - Dizziness and giddiness	4	0
R827 - Abnormal findings in urine, microbiological findings	4	0
T793 - Post-traumatic wound infection, not elsewhere classified	4	0
T846 - Infection and inflammatory reaction due to internal fixation device, hip and thigh	4	0
T846 - Infection and inflammatory reaction due to internal fixation device, knee and lower leg	4	0
T846 - Infection and inflammatory reaction due to internal fixation device, unspecified site	4	0
T855 - Mechanical complication of other gastrointestinal prosthetic devices, implants and grafts	4	0
B023 - Disseminated zoster	3	0
C160 - Malignant neoplasm of cardia	3	0
C649 - Malignant neoplasm of unspecified part of uterus	3	0
C679 - Malignant neoplasm of uterus, part unspecified	3	0
D381 - Neoplasm of uncertain behavior of pancreas	3	0
D860 - Sarcoidosis of lung	3	0
E101 - Non-insulin-dependent diabetes mellitus, with ketoacidosis	3	0
E115 - Type 1 diabetes mellitus, with other specified complications	3	0
E871 - Adverse effect of antineoplastic and immunosuppressive drugs, initial encounter	3	0
F059 - Delirium, unspecified	3	0
G409 - Epilepsy, unspecified	3	0
I214 - Non-ST elevation (NSTEMI) myocardial infarction	3	0
I219 - Acute myocardial infarction, unspecified	3	0

I634 - Cerebral infarction due to unspecified occlusion or stenosis of cerebral arteries	3	0
I802 - Phlebitis and thrombophlebitis of superficial vessels of unspecified lower extremity	3	0
I951 - Hypertension secondary to other renal disorders	3	0
J039 - Acute tonsillitis, unspecified	3	0
J205 - Acute bronchitis due to unspecified organism	3	0
K358 - Other specified diseases of anus and rectum	3	0
K521 - Abscess of anal canal	3	0
K579 - Diverticulitis of colon without perforation or abscess	3	0
K611 - Anal fissure, chronic	3	0
K631 - Anal fistula	3	0
K750 - Pilonidal sinus with abscess	3	0
L023 - Cutaneous abscess, furuncle and carbuncle of buttock	3	0
M002 - Other acute infections of finger	3	0
M009 - Local infection of unspecified site	3	0
M549 - Dorsalgia, unspecified	3	0
N151 - Renal hypertension	3	0
N185 - Chronic kidney disease, stage 5	3	0
N200 - Calculus of kidney	3	0
R104 - Abdominal pain unspecified	3	0
R104 - Other and unspecified abdominal pain	3	0
R400 - Somnolence	3	0
R519 - Headache	3	0
R748 - Abnormal levels of other specified serum enzymes	3	0
R919 - Abnormal radiological findings in lung	3	0
Z038 - Observation for suspected diseases and conditions ruled out	3	0
Z038 - Observation for suspected infectious disease (bacterial, viral) ruled out	3	0
Z038 - Observation for suspected other specified disease ruled out	3	0
Z768 - Other specified contact with health services	3	0
A402 - Sepsis due to streptococcus, group D	2	0
A408 - Other streptococcal sepsis	2	0
A879 - Viral and unspecified gastroenteritis and colitis	2	0
C155 - Malignant neoplasm of intrahepatic bile duct	2	0
C239 - Malignant neoplasm of gallbladder, unspecified	2	0
C787 - Secondary malignant neoplasm of liver and intrahepatic bile duct	2	0
C830 - Malignant neoplasm of adrenal gland	2	0
C851 - Malignant neoplasm of head of pancreas	2	0
C911 - Chronic lymphocytic leukemia of B-cell type	2	0
D462 - Refractory anemia with excess of blasts	2	0
D591 - Hyperproteinemia, unspecified	2	0
D709 - Coagulation defect, unspecified	2	0
E109 - Non-insulin-dependent diabetes mellitus, without complications	2	0
E111 - Type 1 diabetes mellitus, with coma	2	0
E439 - Unspecified disorder of adrenal gland	2	0
E875 - Adverse effect of other drugs, medicaments and biological substances, initial encounter	2	0

G419 - Status epilepticus, unspecified	2	0
G459 - Transient cerebral ischemic attack, unspecified	2	0
G610 - Guillain-Barre syndrome	2	0
I389 - Other disorders of the pericardium	2	0
I480 - Paroxysmal atrial fibrillation	2	0
I619 - Intracerebral hemorrhage, unspecified	2	0
I633 - Cerebral infarction due to embolism of cerebral arteries	2	0
I808 - Phlebitis and thrombophlebitis of other sites	2	0
J009 - Acute nasopharyngitis (common cold), unspecified	2	0
J153 - Pneumonia due to Staphylococcus aureus	2	0
J157 - Pneumonia in diseases classified elsewhere	2	0
J180 - Bronchopneumonia, unspecified	2	0
J188 - Bronchopneumonia, unspecified	2	0
J204 - Acute bronchitis due to other specified organisms	2	0
J851 - Acute pulmonary edema	2	0
K112 - Acute gastric ulcer with perforation	2	0
K121 - Acute duodenal ulcer with hemorrhage	2	0
K264 - Duodenal ulcer, unspecified as acute or chronic, without hemorrhage or perforation	2	0
K403 - Unspecified umbilical hernia with obstruction, without gangrene	2	0
K550 - Nonspecific acute colitis	2	0
K768 - Other specified diseases of anus and rectum	2	0
K831 - Chronic pancreatitis	2	0
K920 - Infectious folliculitis	2	0
L024 - Cutaneous abscess, furuncle and carbuncle of limb	2	0
L893 - Acanthosis nigricans	2	0
M008 - Other specified local infections of skin and subcutaneous tissue	2	0
M139 - Monoarthritis, unspecified	2	0
M359 - Disorder of joint, unspecified	2	0
M628 - Other specified disorders of muscle (acquired)	2	0
M791 - Myalgia	2	0
M866 - Other chronic osteomyelitis	2	0
M870 - Osteonecrosis due to previous trauma, unspecified	2	0
N130 - Hydronephrosis with renal and ureteral calculous obstruction	2	0
N133 - Renal and perinephric abscess	2	0
N184 - Chronic kidney disease, stage 4	2	0
N321 - Vesicoureteral-reflux without reflux nephropathy	2	0
R189 - Ascites	2	0
R559 - Fainting and collapse	2	0
R589 - Hemorrhage, not elsewhere classified	2	0
R700 - Elevated erythrocyte sedimentation rate	2	0
S009 - Superficial injury of head, unspecified	2	0
S320 - Closed fracture of lumbar spine	2	0
T813 - Rupture of operation wound, not elsewhere classified	2	0
T817 - Vascular complications following a procedure, not elsewhere classified	2	0

T831 - Mechanical complication of other internal prosthetic devices, implants and grafts in urinary tract	2	0
T853 - Mechanical complication of other ocular prosthetic devices, implants and grafts	2	0
T886 - Anaphylactic shock due to correct substance properly administered	2	0
A042 - Enteroinvasive Escherichia coli infection	1	0
A043 - Enterohemorrhagic Escherichia coli infection	1	0
A049 - Bacterial intestinal infection, unspecified	1	0
A090 - Other and unspecified gastroenteritis and colitis of infectious origin	1	0
A269 - Mumps meningitis	1	0
A280 - Other viral encephalitis, not elsewhere classified	1	0
A318 - Other specified viral diseases of central nervous system	1	0
A327 - Yellow fever with other organ involvement	1	0
A329 - Yellow fever, unspecified	1	0
A409 - Streptococcal sepsis, unspecified	1	0
A412 - Septicemia due to unspecified staphylococcus	1	0
A413 - Septicemia due to anaerobes	1	0
B003 - Chickenpox with other complications	1	0
B009 - Chickenpox without complications	1	0
B021 - Zoster encephalitis	1	0
B269 - Mumps orchitis	1	0
B271 - Mumps nephritis	1	0
B279 - Mumps without complication	1	0
B377 - Candidal sepsis	1	0
B378 - Candidiasis of other urogenital sites	1	0
C021 - Malignant neoplasm of rectosigmoid junction	1	0
C180 - Malignant neoplasm of cecum	1	0
C182 - Malignant neoplasm of ascending colon	1	0
C187 - Malignant neoplasm of overlapping sites of colon	1	0
C188 - Malignant neoplasm of other parts of colon	1	0
C209 - Malignant neoplasm of colon, unspecified	1	0
C220 - Liver cell carcinoma	1	0
C221 - Intrahepatic bile duct carcinoma	1	0
C240 - Malignant neoplasm of extrahepatic bile ducts	1	0
C241 - Malignant neoplasm of ampulla of Vater	1	0
C248 - Malignant neoplasm of overlapping sites of biliary tract	1	0
C310 - Malignant neoplasm of nasopharynx	1	0
C431 - Malignant melanoma of other parts of face	1	0
C444 - Malignant neoplasm of skin of scalp and neck	1	0
C509 - Malignant neoplasm of unspecified part of the uterus	1	0
C569 - Malignant neoplasm of unspecified part of the colon	1	0
C678 - Malignant neoplasm of other specified parts of uterus	1	0
C711 - Malignant neoplasm of frontal lobe	1	0
C712 - Malignant neoplasm of temporal lobe	1	0
C713 - Malignant neoplasm of parietal lobe	1	0
C740 - Malignant neoplasm of cerebral ventricle	1	0

C762 - Malignant neoplasm of cerebellum	1	0
C780 - Secondary malignant neoplasm of lung	1	0
C786 - Secondary malignant neoplasm of retroperitoneum and peritoneum	1	0
C792 - Secondary malignant neoplasm of central nervous system	1	0
C798 - Secondary malignant neoplasm of other specified sites	1	0
C812 - Kaposi's sarcoma of skin	1	0
C819 - Kaposi's sarcoma, unspecified	1	0
C910 - Acute lymphoblastic leukemia	1	0
C925 - Chronic myeloid leukemia, BCR/ABL-positive	1	0
C931 - Monocytic leukemia	1	0
D375 - Neoplasm of uncertain behavior of rectum	1	0
D410 - Benign neoplasm of rectum	1	0
D412 - Benign neoplasm of pancreas	1	0
D487 - Neoplasm of uncertain behavior of bone and articular cartilage	1	0
D508 - Other specified neoplasms of uncertain behavior of digestive organs	1	0
D611 - Polycythemia vera	1	0
D696 - Thrombocytopenia, unspecified	1	0
D861 - Sarcoidosis of lymph nodes	1	0
E049 - Unspecified thyroiditis	1	0
E055 - Thyrotoxicosis with toxic multinodular goiter	1	0
E106 - Non-insulin-dependent diabetes mellitus, with other specified complications	1	0
E107 - Non-insulin-dependent diabetes mellitus, with multiple complications	1	0
E110 - Type 1 diabetes mellitus, with ketoacidosis	1	0
E119 - Type 1 diabetes mellitus, without complications	1	0
E222 - Acromegaly and pituitary gigantism	1	0
E271 - Primary adrenocortical insufficiency	1	0
E272 - Other adrenocortical insufficiency	1	0
E274 - Ectopic ACTH syndrome	1	0
E859 - Other specified adverse effects, not elsewhere classified	1	0
E870 - Adverse effect of antineoplastic and immunosuppressive drugs, unspecified	1	0
F039 - Unspecified dementia	1	0
F103 - Mental and behavioral disorders due to use of alcohol, withdrawal state with delirium	1	0
F199 - Mental disorder, unspecified	1	0
F312 - Mixed anxiety and depressive disorder	1	0
G000 - Hemophilus meningitis	1	0
G001 - Pneumococcal meningitis	1	0
G002 - Streptococcal meningitis	1	0
G019 - Viral meningitis, unspecified	1	0
G048 - Other encephalitis, myelitis and encephalomyelitis	1	0
G049 - Encephalitis, myelitis and encephalomyelitis, unspecified	1	0
G061 - Intracranial hypertension	1	0
G209 - Alzheimer's disease, unspecified	1	0
G218 - Other specified degenerative diseases of nervous system	1	0
G239 - Unspecified degenerative disease of nervous system	1	0

G319 - Degenerative disease of nervous system, unspecified	1	0
G408 - Other epilepsy	1	0
G708 - Other disorders of myoneural junction and muscle	1	0
H660 - Otitis media, unspecified	1	0
I260 - Pulmonary embolism with acute cor pulmonale	1	0
I313 - Pericarditis, unspecified	1	0
I350 - Aortic valve stenosis	1	0
I426 - Nonrheumatic pulmonary valve stenosis	1	0
I440 - Atrioventricular and left bundle-branch block	1	0
I495 - Sudden cardiac death, so described	1	0
I609 - Intracranial hemorrhage, unspecified	1	0
I612 - Subdural hemorrhage following injury without mention of open intracranial wound, unspecified	1	0
I791 - Septic shock	1	0
I819 - Phlebitis and thrombophlebitis of unspecified site	1	0
I828 - Embolism and thrombosis of other veins	1	0
I970 - Postprocedural hypertension	1	0
J010 - Acute sinusitis, maxillary	1	0
J051 - Acute laryngitis	1	0
J110 - Influenza with pneumonia, virus not identified	1	0
J111 - Influenza with other respiratory manifestations, virus not identified	1	0
J122 - Swine influenza, due to identified swine influenza virus	1	0
J123 - Pandemic influenza, virus not identified	1	0
J201 - Acute bronchitis due to Haemophilus influenzae	1	0
J208 - Acute bronchitis due to other specified organisms	1	0
J210 - Acute bronchiolitis due to respiratory syncytial virus	1	0
J329 - Chronic sinusitis, unspecified	1	0
J390 - Acute pharyngitis, unspecified	1	0
J409 - Acute laryngitis, unspecified	1	0
J449 - Chronic obstructive pulmonary disease, unspecified	1	0
J451 - Simple chronic bronchitis	1	0
J459 - Chronic bronchitis, unspecified	1	0
J479 - Bronchiectasis, unspecified	1	0
J702 - Acute drug-induced interstitial lung disorders	1	0
J849 - Other specified interstitial pulmonary diseases	1	0
J969 - Respiratory failure, unspecified	1	0
J980 - Acute bronchospasm	1	0
J986 - Acute respiratory distress syndrome	1	0
K047 - Chronic pancreatitis	1	0
K052 - Toxic liver disease with hepatic necrosis	1	0
K122 - Acute duodenal ulcer with perforation	1	0
K219 - Acute gastric ulcer, unspecified as acute or chronic, without hemorrhage or perforation	1	0
K226 - Acute duodenal ulcer, unspecified as acute or chronic, without hemorrhage or perforation	1	0
K250 - Gastric ulcer, unspecified as acute or chronic, without hemorrhage or perforation	1	0

K252 - Duodenal ulcer, unspecified as acute or chronic, without hemorrhage or perforation	1	0
K263 - Gastric ulcer, unspecified as acute or chronic, without hemorrhage or perforation	1	0
K297 - Functional disorders of stomach	1	0
K352 - Hypertrophy of anal papillae	1	0
K353 - Fissure and fistula of anal canal	1	0
K500 - Crohn's disease of small intestine	1	0
K519 - Crohn's disease of unspecified site	1	0
K520 - Fistula of anal canal	1	0
K528 - Other specified diseases of anus and rectum	1	0
K566 - Anal sphincter tear, not elsewhere classified	1	0
K658 - Other specified diseases of anus and rectum	1	0
K661 - Ulcer of anal canal	1	0
K808 - Other cholecystitis	1	0
K811 - Chronic cholecystitis with obstruction	1	0
K818 - Other cholecystitis	1	0
K852 - Recurrent appendicitis	1	0
L022 - Cutaneous abscess, furuncle and carbuncle of foot	1	0
L038 - Cellulitis of other specified sites	1	0
L039 - Cellulitis, unspecified	1	0
L050 - Pilonidal cyst with abscess	1	0
L088 - Other specified local infections of skin and subcutaneous tissue	1	0
L120 - Bullous pemphigoid	1	0
L270 - Cutaneous mastocytosis	1	0
L889 - Other specified disorders of skin and subcutaneous tissue	1	0
L892 - Granuloma annulare	1	0
L958 - Other specified reactions to unspecified agents	1	0
M029 - Acute lymphadenitis, unspecified	1	0
M058 - Other infective arthritis	1	0
M069 - Arthritis, unspecified	1	0
M119 - Juvenile arthritis, unspecified	1	0
M130 - Polyarthritis, unspecified	1	0
M161 - Other specified arthritis, knee	1	0
M250 - Other joint disorders, not elsewhere classified, shoulder region	1	0
M259 - Joint disorder, unspecified	1	0
M329 - Juvenile osteochondrosis of spine, unspecified	1	0
M351 - Other hypertrophy of bone (acquired)	1	0
M463 - Spondylopathy, unspecified	1	0
M464 - Vertebrogenic disorders of back, not elsewhere classified	1	0
M465 - Low back pain	1	0
M480 - Spinal stenosis, unspecified	1	0
M485 - Other current conditions in the musculoskeletal system and connective tissue, specified	1	0
M542 - Cervicalgia	1	0
M544 - Lumbago with sciatica	1	0

M600 - Deformity of finger(s), acquired	1	0
M651 - Other synovitis and tenosynovitis, hand	1	0
M704 - Other soft tissue disorders related to use, overuse and pressure, shoulder region	1	0
M706 - Other soft tissue disorders related to use, overuse and pressure, thigh	1	0
M711 - Longitudinal tear of medial meniscus, current	1	0
M726 - Other bursopathies, not elsewhere classified	1	0
M792 - Neuralgia and neuritis, unspecified	1	0
M799 - Soft tissue disorder, unspecified	1	0
M860 - Acute hematogenous osteomyelitis, unspecified	1	0
M966 - Osteoporosis, unspecified	1	0
N049 - Unspecified nephritis, nephritic syndrome, and nephrosis	1	0
N131 - Hydronephrosis with other obstructive uropathy	1	0
N132 - Hydronephrosis, unspecified	1	0
N178 - Other specified renal tubulo-interstitial diseases	1	0
N183 - Chronic kidney disease, stage 3	1	0
N450 - Epididymitis	1	0
N491 - Inflammatory diseases of uterus, except cervix	1	0
N492 - Inflammatory disease of cervix uteri	1	0
N619 - Inflammatory disease of ovary, fallopian tube, and broad ligament, unspecified	1	0
O080 - Incomplete spontaneous abortion with other and unspecified complications	1	0
O860 - Other specified complications of labor and delivery, delivered, with or without mention of antepartum condition	1	0
O911 - Venous complication in the puerperium	1	0
O991 - Diseases of the blood and blood-forming organs and certain disorders involving the immune mechanism complicating pregnancy, childbirth, and the puerperium	1	0
R059 - Cough	1	0
R068 - Other and unspecified abnormalities of breathing	1	0
R093 - Abnormal sputum	1	0
R098 - Other specified symptoms and signs involving the circulatory and respiratory systems	1	0
R238 - Other specified skin changes	1	0
R296 - Predisposition to falling, not elsewhere classified	1	0
R298 - Other and unspecified symptoms and signs involving the nervous and musculoskeletal systems	1	0
R401 - Stupor	1	0
R402 - Coma, unspecified	1	0
R529 - Pain, unspecified	1	0
R568 - Seizures, unspecified	1	0
R591 - Generalized lymphadenopathy	1	0
R600 - Edema of lower limb, unspecified	1	0
R798 - Other specified abnormal findings of blood chemistry	1	0
S008 - Superficial injury of other specified parts of head	1	0
S220 - Closed fracture of sternum	1	0
S224 - Multiple fractures of ribs	1	0
S300 - Contusion of lower back and pelvis	1	0

S328 - Fracture of other and unspecified parts of lumbar spine and pelvis	1	0
S423 - Fracture of shaft of humerus, open	1	0
S519 - Open wound of forearm, unspecified	1	0
S525 - Fracture of lower end of radius	1	0
S721 - Pertrochanteric fracture, closed	1	0
S730 - Dislocation of hip	1	0
S800 - Contusion of knee	1	0
S910 - Open wound of ankle	1	0
S911 - Open wound of toe(s) without damage to nail	1	0
T181 - Foreign body in esophagus	1	0
T243 - Burn of third degree of hip and lower limb, except ankle and foot	1	0
T782 - Anaphylactic shock, unspecified	1	0
T783 - Angioneurotic edema	1	0
T801 - Complication of infusion, transfusion and therapeutic injection	1	0
T802 - Infection following infusion, transfusion and therapeutic injection	1	0
T810 - Bleeding and hematoma complicating a procedure, not elsewhere classified	1	0
T812 - Accidental puncture and laceration during a procedure, not elsewhere classified	1	0
T828 - Other specified complications of cardiac and vascular prosthetic devices, implants and grafts	1	0
T830 - Mechanical complication of other vascular grafts	1	0
T832 - Mechanical complication of other urinary devices and implants	1	0
T834 - Mechanical complication of other internal orthopedic devices, implants and grafts	1	0
T840 - Mechanical complication of internal joint prosthesis of hip	1	0
T856 - Mechanical complication of other specified internal prosthetic devices, implants and grafts	1	0
T858 - Other specified complications of internal prosthetic devices, implants and grafts, not elsewhere classified	1	0
T887 - Unspecified adverse effect of drug or medicament	1	0
T888 - Other specified complications of surgical and medical care, not elsewhere classified	1	0
U072 - COVID-19, virus not identified	1	0
U129 - COVID-19 vaccines in therapeutic use as the cause of adverse effects	1	0
Z867 - Personal history of other diseases and conditions	1	0
