# Supplementary material for: The performance of screening tools and use of blood analyses in prehospital identification of sepsis patients and patients suitable for non-conveyance - an observational study
Source: BMC Emerg Med. 2024 Oct 8;24:180. doi: 10.1186/s12873-024-01098-4 (PMC11462654; doi:10.1186/s12873-024-01098-4)
Supplement: Supplementary file 4 — Supplementary Material 4 Additional file 4 Forest plot of screening tool accuracy [file 12873_2024_1098_MOESM4_ESM.pdf]

Additional file 4 – Forest plot of screening tool accuracy

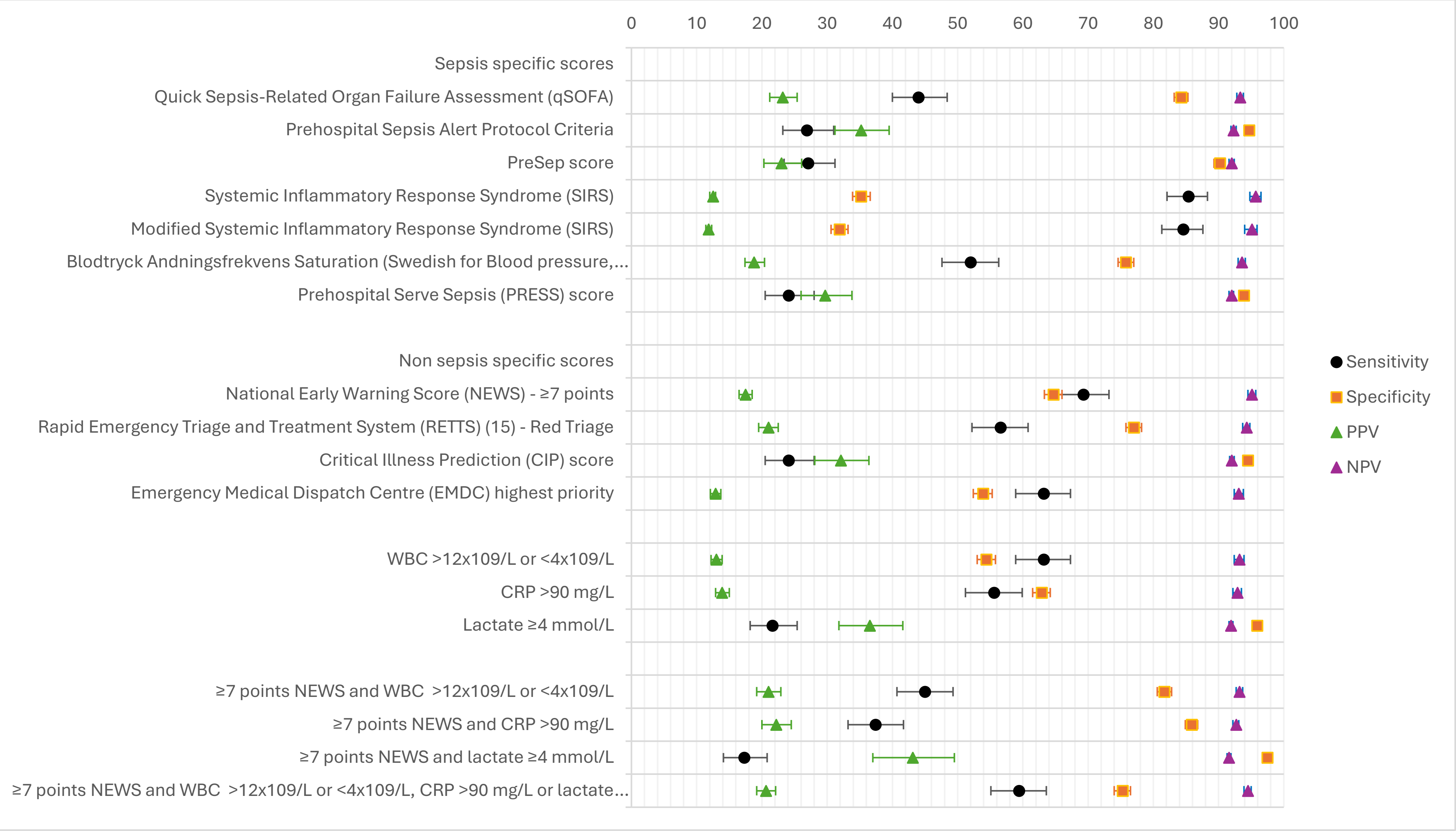

PPV = predictive value, NPV = Negative predictive value, WBC = White Blood Cell Count, CRP = C-Reactive Protein
